# Assessment of the feasibility of exon 45–55 multiexon skipping for duchenne muscular dystrophy

**DOI:** 10.1186/1471-2350-9-105

**Published:** 2008-12-01

**Authors:** Laura van Vliet, Christa L de Winter, Judith CT van Deutekom, Gert-Jan B van Ommen, Annemieke Aartsma-Rus

**Affiliations:** 1 DMD Genetic Therapy Group, Department of Human Genetics, Leiden University, Medical Center, Postzone S4-P, PO Box 9600, 2300RC, Leiden, The Netherlands; 2 Prosensa Therapeutics B.V, Wassenaarseweg 72, 2333AL, Leiden, The Netherlands

## Abstract

**Background:**

The specific skipping of an exon, induced by antisense oligonucleotides (AON) during splicing, has shown to be a promising therapeutic approach for Duchenne muscular dystrophy (DMD) patients. As different mutations require skipping of different exons, this approach is mutation dependent. The skipping of an entire stretch of exons (e.g. exons 45 to 55) has recently been suggested as an approach applicable to larger groups of patients. However, this multiexon skipping approach is technically challenging. The levels of intended multiexon skips are typically low and highly variable, and may be dependent on the order of intron removal. We hypothesized that the splicing order might favor the induction of multiexon 45–55 skipping.

**Methods:**

We here tested the feasibility of inducing multiexon 45–55 in control and patient muscle cell cultures using various AON cocktails.

**Results:**

In all experiments, the exon 45–55 skip frequencies were minimal and comparable to those observed in untreated cells.

**Conclusion:**

We conclude that current state of the art does not sufficiently support clinical development of multiexon skipping for DMD.

## Background

Antisense-mediated exon skipping is emerging as a very promising therapeutic approach for Duchenne muscular dystrophy (DMD) [1]. The aim of this approach is to restore the disrupted reading frame of *DMD *transcripts, and allow synthesis of partly functional, internally deleted Becker-like dystrophins, rather than prematurely truncated non-functional Duchenne dystrophins. This can be achieved by antisense oligonucleotides (AONs) that target specific exons and hide them from the splicing machinery during pre-mRNA splicing, resulting in the skipping of said exons [2]. Proof of concept of this strategy has been obtained in numerous patient-derived cell cultures with different types of mutations, the *mdx *mouse model and recently in a first clinical trial where AONs were injected locally in the *tibialis anterior *muscle of 4 Duchenne patients [1,3-9]. One of the disadvantages of this therapy is its mutation specificity: different exons have to be skipped to restore the reading frame for different mutations [10]. Fortunately, most mutations involve deletions of one or more exons between exon 45 and 53 or between exon 2–20 (50% and 15% of all mutations, respectively) [11]. Therefore, restoration of the reading frame for over 50% of all patients (75% of deletion patients) is theoretically feasible using a strategically selected set of only 10 exons [12]. Skipping of exon 51 is beneficial for the largest group of patients (19% of all deletions, or 13% of all Duchenne patients, (Aartsma-Rus *et al*. accepted manuscript).

Nevertheless, it would be more straightforward if a single formulation of AONs would be applicable to an even larger group of patients. To achieve this, we and others suggested skipping of a stretch of consecutive exons simultaneously (multiexon skipping) [4,13]. An added advantage of multiexon skipping is that it allows artificial induction of deletions known to be associated with mild phenotypes. We previously reported the feasibility of skipping a stretch of exons 45 through 51 (applicable to 13% of Duchenne patients) after targeting only the two outer exons with a mix of two AONs [4]. Multiexon skipping levels could be increased by combining the two individual AONs in one molecule [4,14]. Alternatively, a cocktail of AONs targeting all individual exons present in the stretch to be skipped can be employed. This has been successfully applied to induce exon 20–26 skipping in the *mdx *mouse model [15]. However, using cocktails to induce the simultaneous skipping of an increasing number of exons is more challenging, due to the increasing occurrence of intermediate splicing products (personal observation and Steve Wilton, personal communication). These out-of-frame splice intermediates in fact dilute the levels of intended multiexon skipping.

**Figure 1:**
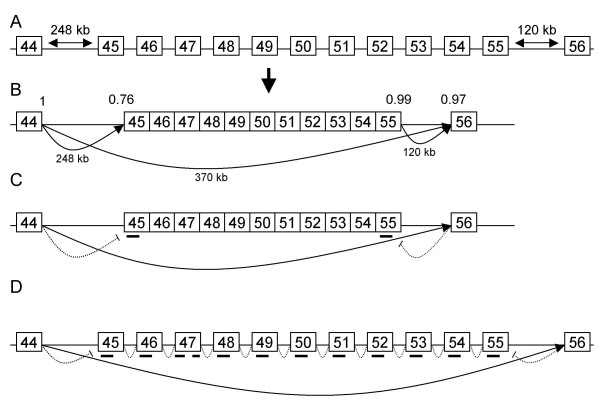
**Antisense-mediated multiexon 45–55 skipping.** A. The introns flanking the intended multiexon skip are extremely large (248 kb and 120 kb for intron 44 and intron 55, respectively), whereas the internal introns are shorter (2.3 – 54 kb). B. It is possible that internal exons are joined before intron 44 is spliced out, resulting in an "exon block" of exons 45–55. Here, the splice acceptors of exons 45 and 56 are competing for the splice donor of exon 44, and the splice donors of exons 44 and 55 are competing for the splice acceptor of exon 56. While the donor sites of exons 44 and 55 are of similar strength (1 and 0.99, respectively, calculated with the Berkely Drosophila Genome Project for human splice site prediction software; ), the acceptor splice site of exon 45 (0.76) is weaker than that of exon 56 (0.97), thus explaining why occasionally exon 44 is joined to exon 56 rather than exon 45. C. Exon 45–55 skipping levels and frequency might be enhanced by AONs targeting exon 45 and exon 55, which should increase the chance that exon 44 and exon 56 are joined. D. Regardless of whether the exon block hypothesis is correct, exon 45–55 skipping can be induced using a cocktail of AONs targeting each of the individual AONs. However, it is more likely that some but not all exons are skipped and that this cocktail gives rise to many intermediate products where one or more (but not all) intended exons are skipped.

We have repeatedly observed that the feasibility of skipping larger stretches of exons is also limited when using AONs targeting the outer exons: anticipated multiexon skipping patterns were not or not reproducibly induced. This is probably due to the fact that *DMD *introns are extremely large and *DMD *pre-mRNA is cotranscriptionally spliced [14]. E.g. inducing exon 17–48 multiexon skipping is (nearly) impossible, as exon 17 is joined to exon 16 long before exon 48 is even transcribed (an estimated 4.5 h later) [2,16]. However, as *DMD *intron sizes vary between 107 bp and 360 kb it is not inconceivable that some smaller downstream introns are spliced out prior to larger upstream exons. The most obvious examples would be intron 44, which is 240 kb. Subsequent introns are shorter, until intron 55, which is 120 kb. If the smaller introns (45 through 54) are indeed spliced out prior to intron 44 and intron 55, this would result in an "exon 45–55 block" (see Figure 1) [14]. This hypothesis is underlined by our earlier finding of spontaneous exon 45–55 skipping in untreated control and patient myotube cultures, indicating that the acceptor splice sites of intron 44 and 55 can compete [14]. The existence of an exon 45–55 block implies that exon 45–55 multiexon skipping may be more easily achieved than other and previously studied multiexon skips [12,14]. Notably, exon 45–55 multiexon skipping would be beneficial for ~30% of Duchenne patients present in the Leiden DMD mutation database [14] and almost 65% of the French UMD database [13]. In addition exon 45–55 deletions are associated with very mild Becker phenotypes and have even been found in asymptomatic individuals [13]. In this report we therefore focus on the feasibility of enhancing exon 45–55 multiexon skipping in patient and control myotube cultures using different approaches. However, we were unable to increase the levels over those observed due to alternative splicing.

## Methods

### AONs

AONs used for this study (Table 1, 2, 3) were h45AON5L, h46AON26L, h47AON2, h47AON5 (exon 47 skipping requires double targeting), h48AON6, h49AON1, h50AON1, h51AON1, h52AON4, h53AON1, h54AON1 and h55AON5. All AONs have been described previously ([17,18] (Heemskerk, accepted manuscript), except for h52AON4 (uuccaacuggggacgccucuguucc) and the linked AON, which consists of previously described AONs h45AON5L and h55AON5 linked by 10 uracil nucleotides. All AONs consist of 2'-O-methyl RNA and contain a full-length phosphorothioate backbone (Eurogentec, Belgium).

**Table 1:** Overview of the results for the exon 45–55 multiexon skipping experiments in control cells

Used AONs	Exon 45–55 skip	NT	Other skips	NT
Exon 45 & 55 (mix)	1/6	0/6	Exon 45–52 skip (1×)	Exon 45–54 skip (1×)
	0/6	1/6		Exon 45–53 skip (1×)
Exon 45 & 55 (linked)	1/6	1/6	Exon 45–51 & 54–55 skip (1×)	Exon 45–51 skip (1×)
	5/5	5/5		Exon 48–55 skip (1×)
Exon 45, 46, 47, 48, 49, 50, 51, 52, 53, 54 & 55	1/6	1/6	Exon 45–47 & 49–55 skip (1×)	Exon 45–51 skip (1×)
	5/5	5/5		Exon 48–55 skip (1×)

**Table 2:** Overview of the results for the exon 45–55 multiexon skipping experiments in deletion exon 46–50 cell cultures

Used AONs	Exon 45–55 skip	NT	Other skips	NT
Exon 45 & 55 (mix)	4/6	4/6		Exon 45–54 skip (1×)
	3/6	1/6		
Exon 45 & 55 (linked)	1/6	4/6		
	6/6	6/6		
Exon 45, 46, 47, 48, 49, 50, 51, 52, 53, 54 & 55	0/6	4/6	Exon 45–54 skip (1×)	
	6/6	6/6		

**Table 3:** Overview of the results for the exon 45–55 multiexon skipping experiments in deletion exon 48–50 cell cultures

Used AONs	Exon 45–55 skip	NT	Other skips	NT
Exon 45 & 55 (mix)	2/6	3/6	Exon 45–52 skip (1×)	Exon 45–53 skip (2×)
Exon 45 & 55 (linked)	2/6	2/6		Exon 45–53 & 55 skip (1×)
	3/6	4/6		
Exon 45, 46, 47, 48, 49, 50, 51, 52, 53, 54 & 55	0/6	2/6	Exon 45–53 skip (1×)	Exon 45–53 & 55 skip (1×)
	2/6	3/6	Exon 45–53 & 55 skip (1×)	

### Myogenic cell cultures and AON transfections

Primary myoblasts from an anonymous human control and two anonymous Duchenne patients, with deletions of exon 48–50 and exon 46–50 respectively [3,4] were cultured and differentiated into myotubes in 6 wells plates, as described [3]. Cultures were transfected with mixtures of 200 nM of each AON, or 100 nM of each AON in the cocktail experiment, and 2.5 μl polyethyleneimine (PEI, Exgen 500, MBI Fermentas) per μg AON for three hours. Each combination was tested in six-plo and for each experiment six untreated wells were used as reference.

### RNA isolation and RT-PCR analysis

RNA was isolated ~28 hours after transfection using RNA-Bee (Campro Scientific) as described elsewhere [3]. RT-PCR analysis, sequence analysis and DNA lab chip analysis (Agilent Technologies) were performed as described [3,4]. Primers used for RT-PCR analysis were designed in exons upstream and downstream of exon 45 and 55, respectively. Additional primers flanking exon 45 and exon 55 were used to confirm successful transfection of individual AONs (sequence on request).

### Western blot analysis

Protein was isolated 48 hours after AON treatment as described elsewhere [4]. Samples were boiled for 5 minutes, loaded on a 4–7% gradient polyacrylamide gel and run overnight at 4°C. Gels were blotted to nitrocellulose BA83 (Whatman, Schleicher & Schuell, Germany) for 6 hours at 4°C. Blots were blocked with 5% non-fat dried milk (Campina Melkunie, the Netherlands) in TBS followed by an overnight incubation with NCL-DYS1 (dilution 1:125, Novocastra, UK) in TBS plus 0.05% Tween20 to detect dystrophin. The fluorescent IRDye 800 CW goat-anti-mouse IgG (dilution 1:5000, Li-Cor, NE, USA) was used as a secondary antibody. Blots were visualized with the Odyssey system and software (Li-Cor, NE, USA).

## Results and discussion

We hypothesized that multiexon 45–55 skipping might be induced or enhanced using different AONs and combination strategies (Figure 1). If the "exon block" hypothesis is correct, the exon 45 and exon 56 splice acceptor sites compete for the splice donor site of exon 44 (Figure 1B). Even though exon 45 is closer to exon 44 than exon 56 (248 kb vs 370 kb), exon 45 has a weaker acceptor site than exon 56 (0.76 vs 0.97, respectively). This might explain why exon 45–55 are occasionally alternatively or aberrantly spliced out. Targeting the two outer exons (exon 45 and exon 55) will increase the chance that exon 44 and exon 56 are joined (Figure 1C). To enhance the chance that both AONs hybridize to the same pre-mRNA transcript we included an AON where AONs targeting exon 45 and exon 55 are linked by 10 uracil nucleotides. Finally, we tested a cocktail of AONs targeting each individual exon from exon 45 to exon 55 (Figure 1D). This should result in exon 45–55 skipping regardless of the exon block hypothesis. However, this was also expected to generate many intermediate products due to the fact that in some transcripts not all exons will be targeted. Different AON chemistries are available [19]. These include phosphorodiamidate morpholinos, which are very hard to transfect in vitro, and locked nucleic acids, which have a high propensity to bind to other locked nucleic acid oligomers, thus making an approach using a combination complicated [19]. We thus used 2'-*O*-methyl phosphorothioate AONs, as these can be transfected at high efficiencies, and efficient AONs for the targeted exons were available.

**Figure 2:**
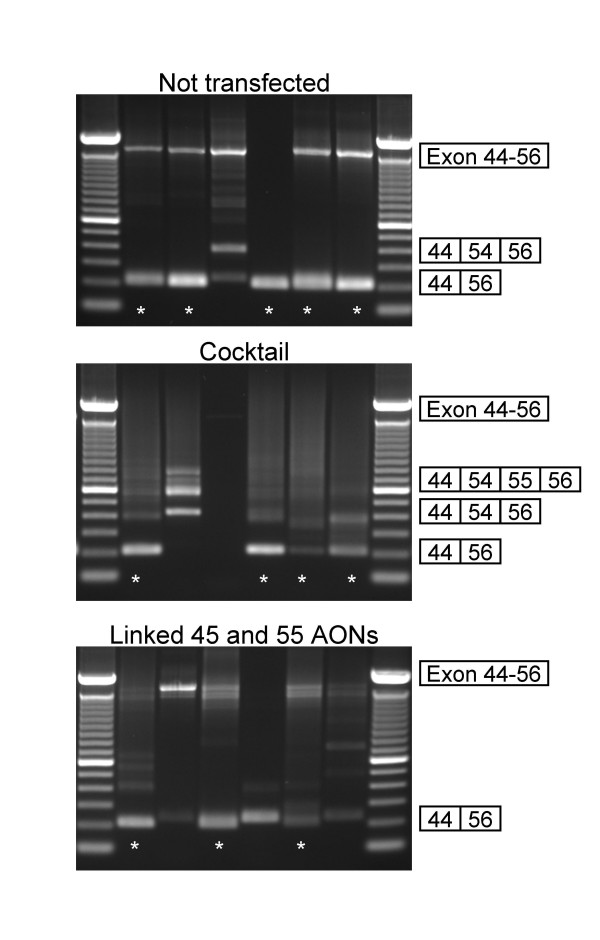
**Example of RT-PCR analysis of exon 45–55 skipping in myotube cultures from an exon 48–50 deletion patient.** Each AON combination was tested in 6 wells and results were compared to 6 untreated wells. Exon 45–55 skipping can be seen in untreated cells (upper panel, lanes 1, 2, 4–6, indicated by a an asterisk) as well as cells treated with the cocktail of AONs (middle panel, lanes 1, 4–6) and the linked AON (lower panel, lanes 1, 3 and 5). In addition, exon 45–53 and 55 skipping (upper panel, lane 3, middle panel, lane 2) and exon 45–53 skipping (middle panel, lane 2) were observed. Additional bands were too faint to allow identification by sequence analysis. The band slightly higher than the 45–55 skip product (e.g. lower panel, lane 4) is a PCR artifact arising from false annealing of somewhat similar sequences in exons 44 and 55. Exon 45–55 skip products were verified by sequencing.

**Figure 3:**
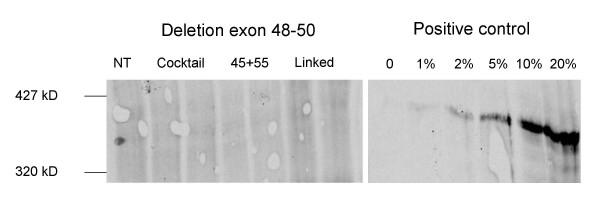
**Western blot analysis of the exon 48–50 deleted cells.** No dystrophin could be observed in non-treated (NT) or treated myotubes. Each treatment was performed in duplo. Myosin staining was used as a loading control and to confirm that myogenicity was sufficient to allow dystrophin expression (data not shown). Different dilutions of protein from control cells were used as a positive control (right panel). Levels of 1% of the normal levels were detectable.

Myotube cultures from a healthy individual and two Duchenne patients were treated with the different combinations of AONs (Table 1, 2, 3) and RNA was isolated on the subsequent day. Each combination was tested in at least six individual samples. As exon 45–55 skipping was previously observed in untreated samples [14] and its frequency varied between different cell batches (Table 1, 2, 3 and data not shown), for each experiment RNA was isolated from 6 untreated samples of the same cell batch as the treated cells. The frequencies of exon 45–55 skipping, as determined by RT-PCR analysis, were compared with and without treatment (Example shown in Figure 2, summary in Table 1, 2, 3). Putative exon 45–55 skipping products were verified by sequencing analysis (data not shown). Unfortunately, similar frequencies were observed, both in control and patient cell cultures, regardless of the combination of AONs used. Efficient transfection was confirmed by RT-PCR focusing on the individually targeted exons (data not shown). In addition to the occasional exon 45–55 skipping we observed several intermediate products, especially after treatment with the AON cocktail (Figure 2 middle panel, Table 1, 2, 3). Note that as the non skipped fragment is relatively large, it was not always amplified. Due to the lack of this reference value, assessment of exon 45–55 skipping levels was difficult. For a number of samples we determined the absolute amount of exon 45–55 skip product, or alternatively, the amount of exon 45–55 skip product relative to the amount of exon 44–45 product (which has a similar length as the skip product, but requires a different reverse PCR primer). In both cases, no differences were observed between multiexon skip levels before and after AON treatment. The exon 45–55 skipping observed after AON treatment is thus most likely due to naturally occurring alternative or aberrant splicing rather than through AON induction. This alternative splicing occurs at very low levels, as confirmed by Western blot analysis where no dystrophin was observed before or after AON treatment of the exon 48–50 deleted cells (Figure 3). Since levels of 1% of wild type dystrophin could be detected, this indicates that if this naturally occurring exon skipping does result in dystrophin production, levels are below 1%.

## Conclusion

We conclude that, despite being theoretically a promising approach, the current state of the art does not sufficiently support clinical development of multiexon 45–55 skipping for DMD. In order to explore the frontiers of multiexon skipping, more information on the order and timing of DMD intron removal is required. Considering the urgent need for therapy, straightforward clinical development of single exon skipping, at this point being reproducible and efficient, should be preferred. In fact, single exon 51 skipping induced by 2'-O-methyl phosphorothioate AON PRO051 is currently in phase I/II clinical trials [1], clinical studies based on single exon 44 skipping trials are being prepared for, and other single exon skipping AONs will follow soon. Although single exon skipping may be applicable for relatively smaller groups of patients, skipping of exons 44, 45, 51 and 53 together would be beneficial to over 50% of all deletion patients, or over 35% of all patients in the Leiden DMD mutation database (Aartsma-Rus *et al*. accepted manuscript).

## Abbreviations

AON: antisense oligonucleotides, DMD: Duchenne muscular dystrophy, UMD: Universal mutation database.

## Competing interests

The authors declare that they have no competing interests.

## Authors' contributions

LvV and CdW performed experiments, JvD and G-JvO helped draft the manuscript and participated in the coordination and design of experiments, AAR performed experiments, conceived of the study, participated in the design and coordination and drafted the manuscript. All authors read and approved of the final manuscript.

## Pre-publication history

The pre-publication history for this paper can be accessed here:


